# Groundwater-Driven Evolution of Prebiotic Alkaline Lake Environments

**DOI:** 10.3390/life14121624

**Published:** 2024-12-07

**Authors:** Benjamin M. Tutolo, Robert Perrin, Rachel Lauer, Shane Bossaer, Nicholas J. Tosca, Alec Hutchings, Serhat Sevgen, Michael Nightingale, Daniel Ilg, Eric B. Mott, Thomas Wilson

**Affiliations:** 1 Department of Earth, Energy, and Environment, University of Calgary, Calgary, AB T2N 1N4, Canada; 2 Department of Earth Sciences, University of Cambridge, Cambridge CB2 3EQ, UK

**Keywords:** alkaline lakes, origin of life, groundwater, phosphorus

## Abstract

Alkaline lakes are thought to have facilitated prebiotic synthesis reactions on the early Earth because their modern analogs accumulate vital chemical feedstocks such as phosphate through the evaporation of dilute groundwaters. Yet, the conditions required for some building block synthesis reactions are distinct from others, and these conditions are generally incompatible with those permissible for nascent cellular function. However, because current scenarios for prebiotic synthesis have not taken account of the physical processes that drive the chemical evolution of alkaline lakes, the potential for the co-occurrence of both prebiotic synthesis and the origins and early evolution of life in prebiotic alkaline lake environments remains poorly constrained. Here, we investigate the dynamics of active, prebiotically relevant alkaline lakes using near-surface geophysics, aqueous geochemistry, and hydrogeologic modeling. Due to their small size, representative range of chemistry, and contrasting evaporation behavior, the investigated, neighboring Last Chance and Goodenough Lakes in British Columbia, Canada offer a uniquely tractable environment for investigating the dynamics of alkaline lake behavior. The results show that the required, extreme phosphate enrichments in alkaline lake waters demand geomorphologically-driven vulnerability to evaporation, while the resultant contrast between evaporated brines and inflowing groundwaters yields Rayleigh–Taylor instabilities and vigorous surface–subsurface cycling and mixing of lake and groundwaters. These results provide a quantitative basis to reconcile conflicting prebiotic requirements of UV light, salinity, metal concentration, and pH in alkaline lake environments. The complex physical and chemical processing inherent to prebiotic alkaline lake environments thus may have not only facilitated prebiotic reaction networks, but also provided habitable environments for the earliest evolution of life.

## 1. Introduction

The nature of the geological processes leading to the origins and early evolution of life on Earth is hotly debated [1]. Alkaline lakes on the surface of early Earth have received widespread support as environmental hosts for prebiotic reaction networks [2,3,4,5] because they would have provided access to feedstocks and conditions required to facilitate selective and high-yielding prebiotic syntheses of several molecular building blocks [6,7,8]. These lakes would have also been inherently prone to evaporation and wet–dry cycling, which are needed for nucleotide synthesis [9]. In turn, evaporative processes would also have led to uniquely elevated concentrations of essential components, most importantly phosphate (at minimum concentrations of 0.1 to 1 mol/kg), required in prebiotic synthesis experiments [6,7,8]. Alkaline lakes thus offer a long-sought solution to the “phosphate problem” in origins studies [3,10].

Nevertheless, the features of alkaline lakes that make them attractive locations for the origin of life may have also posed challenges. For instance, the evaporation processes that yield elevated phosphate concentrations also increase the concentration of many other solutes. While this elevated salinity can drive condensation and polymerization reactions through its effect on water activity [11], it may inhibit the formation of some lipid membranes [12], promote the synthesis of others [13,14], and negatively impact cell viability [15]. Moreover, concentrations of metal ions exhibit variable effects on peptide synthesis and stability [16], fatty acid vesicle formation [17], and RNA structure and function [18]. While fluctuations in pH can be essential for all of these processes [19,20], the exceptionally well-buffered nature of alkaline lake solutions works to strongly fix pH [21]. Finally, while UV light transmitted through the sun-lit surface of alkaline lakes may be important for prebiotic chemistry [22]—e.g., it serves as a source of energy and selectivity in the reductive homologation of HCN [8] and in CO_2_ reduction and subsequent carboxysulfitic chemistry [23]—its effects can also be destructive, because several intermediates formed during these pathways are susceptible to photolysis [24,25]. Recent work suggests that alkaline lake waters are permissive of RNA and cell membrane functionality, although only after adjustments to pH and/or freshening events [26].

When viewed together, the physical and chemical features of alkaline lakes challenge their plausibility as original environments. Yet, it is well established that proposed prebiotic reaction networks in fact require the evolution of chemistry and environmental conditions to produce prebiotic building blocks, i.e., few expect that all reactions could truly proceed in a single, isolated system such as an alkaline lake pool [27]. Because virtually all of our knowledge of alkaline lakes in the context of the origin of life stems from individual analyses of evolved lake water chemistry decoupled from the physical processes that shape the lake system overall, it is unclear whether alkaline lake systems contain interconnected sub-environments that, together, would permit the full suite of prebiotic reaction network branches to proceed in tandem. To this end, the link between alkaline lake water chemistry and the chemistry of dilute local groundwater suggests that these systems are indeed characterized by a range of salinities, metal concentrations, pH, and UV exposures. However, without the focused study of the physical and chemical processes and timescales inherent in generating and sustaining alkaline lakes, and in endowing them with prebiotically relevant properties, we cannot know whether alkaline lakes could feasibly support life’s origins.

**Figure 1:**
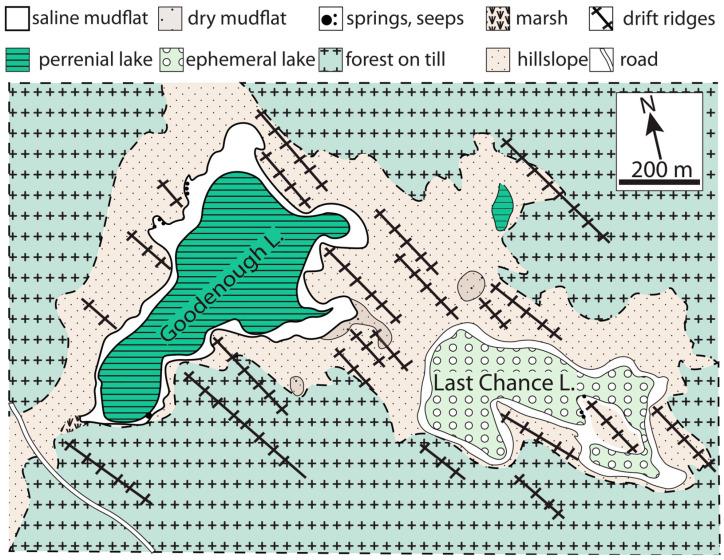
Site map of the study area, showing Last Chance and Goodenough Lakes and associated sub-environments. Figure redrawn from Renaut and Long [28].

Here, we evaluate the hypothesis that groundwater–lake water interactions in alkaline lakes can provide a solution to the paradoxical observation that the conditions required for certain branches of prebiotic networks are incompatible with others and those permissible for nascent cellular function. To do this, we couple near-surface geophysical surveys with analytical geochemistry and hydrogeological modeling to investigate the groundwater–lake water interactions underpinning the development of two adjacent, uniquely phosphorus-rich lakes, Last Chance Lake and Goodenough Lake, located in British Columbia, Canada (Figure 1). This approach allows us to identify controls on extreme phosphate accumulation and reveals unexpected dynamic physical behavior that carries numerous implications for prebiotic synthesis that are also characteristic of saline lake systems more generally.

## 2. Materials and Methods

### 2.1. Geological Context for the Study Site

Alkaline lakes are unique geochemical environments that occur where required hydrologic conditions and catchment geology converge [29]. Due to their small size, representative range of P concentrations, and contrasting evaporation behavior, Last Chance and Goodenough Lakes in British Columbia, Canada offer a uniquely tractable opportunity for investigating the dynamics of alkaline lake behavior. The two lakes are separated by a ca. 200 m wide glacial sediment ridge (Figure 1), with Goodenough Lake slightly (~2 m) higher in elevation and slightly larger (maximum ~0.18 km^2^) than Last Chance Lake (maximum 0.13 km^2^) [30]. Beginning approximately a century ago [31], these two lakes have been extensively studied in the context of alkaline lake chemical sedimentation e.g., [28,29,30,31,32,33,34] and biological habitability and ecology [10,29,35,36,37,38]. Following the discovery by Toner and Catling [3], on the basis of chemical analyses reported by Hirst [30], that Last Chance Lake contains the highest P concentrations of any known natural water, the origins of life community has begun to concentrate research on these and other alkaline lake systems globally [10,26,39]. Building on this previous work, we analyzed the chemical and hydrologic properties of the lakes and their subsurface structure and composition using the suite of techniques described in this section.

### 2.2. Chemical Analyses

Lake water and spring water samples were collected in polyethylene bottles or tubes that were first rinsed in the sampled water before taking the sample for processing. Groundwaters were obtained via a plastic, ball valve-capped bailer inserted into stainless steel (DP1 and DP3) or steel (DP 2 and DP4) pipes outfitted with Solinst^®^ (Solinst Canada Ltd., Georgetown, ON, Canada) Drive-Point piezometers driven below the water table into the subsurface (typical sampling depths are 1–1.5 m). We also extracted four sediment cores from Last Chance Lake in June 2021 at varying distances from the site of observed groundwater springs, assumed to be a locus of groundwater input to the lake. Porewaters were extracted from sediment cores at 2 cm intervals below the sediment–water interface and analyzed for alkalinity, pH, and phosphate concentrations. Full details of water sample acquisition and geochemical analyses are presented in Section S1. To evaluate the evolution of water chemistry as groundwaters evolve to lake waters, the solubility of hydroxylapatite in Na-HCO_3_ solutions in equilibrium with an atmosphere containing 10^−3.5^ bars of CO_2_ at 25 °C was determined using the Geochemist’s Workbench version 17.03.3 (Aqueous Solutions, LLC (Champaign, IL, USA)) React module outfitted with the THEREDA 2021 thermodynamic database. The hydroxylapatite data included in this database were evaluated and selected by the Thermodynamic Reference Database (THEREDA) project through a rigorous process described by Scharge [40]. Evaporation was simulated by adding Na^+^ to an initially dilute, calcite- and hydroxylapatite-equilibrated open with respect to atmospheric CO_2_. As Na^+^ increased, Ca^2+^ precipitated as calcite. Maximum calculated hydroxylapatite solubility and phosphate concentration were achieved whenever the solution became supersaturated with respect to natron; after this point, additional Na^+^ addition to the atmospherically equilibrated solution simply yielded further natron precipitation. While this approach ignores the intricacies of the chemistry of the alkaline lake brine and the potential effects of temperature fluctuations on mineral solubility, it produces remarkably accurate predictions of maximum phosphate concentrations as a function of alkalinity because, in the aggregate, these other solutes have a negligible effect on solution alkalinity (see charge balances presented in Data Set S1).

### 2.3. Near-Surface Geophysics

To evaluate the physical properties of the subsurface underlying and between Last Chance and Goodenough Lakes, seismic refraction tomography data were acquired using a Geometrics Geode acquisition system with a total of 60 single-component geophones spaced at 2.5 m and surveyed via a Benchmark Hemisphere S321 GPS unit with real-time kinematic corrections. A sledgehammer was used as the seismic source and shots were taken at 5 m intervals. The resultant data were inverted using the Rayfract version 3.3.5 software package, which uses Wavepath Eikonal Traveltime Inversion e.g., ref. [41] to estimate subsurface seismic velocities. This inversion method was chosen over a layer-based analysis method e.g., ref. [42] because of its ability to address some of the shortcomings of a layer-based method (i.e., pinch-outs and outcrops) which were possible at this site. Nevertheless, tomographic methods, such as the one used here, are subject to limitations in environments where there is a sharp vertical velocity contrast (as was expected at the sediment/basalt interface at this site) because of their tendency to overly smooth the output model to avoid producing artifacts. We addressed this limitation by using a layer-based starting velocity model with a narrow wavepath width in the tomographic inversion, following the method described by Rohdewald [43], which allowed us to reduce smoothing at the sediment/basalt interface, while reducing the introduction of inversion artifacts in the resulting velocity model.

To visualize freshwater input zones, saline water circulation depths, and spatial interactions between saline brines and fresh groundwaters in the subsurface underlying Last Chance and Goodenough Lakes, we used Electrical Resistivity Tomography (ERT) and a complementary, surface-based electromagnetic (EM) technique. Four ERT surveys were performed at locations around Last Chance Lake in October 2019 and three surveys were acquired around Goodenough and Last Chance in October 2021 (Table S2) using an ABEM Terrameter LS 2 and electrode locations surveyed using a Benchmark Hemisphere S321 GPS unit with real-time kinematic corrections. Absolute values of elevation were referenced to a location between Last Chance and Goodenough Lakes, ensuring that all measured locations are highly precise in relation to one another and therefore suitable for the purposes of this study. Nevertheless, the presented elevations deviate from recorded elevations of ~1080 m [30] and should thus be treated accordingly. These surveys occurred after a summer of typical rainfall and temperature conditions (2019) and after a particularly warm and dry summer (2021) (Figures S1–S4). Each line consisted of 81 electrodes, with differing electrode spacings and line lengths (Table S1). Line 4 was acquired “on top of” line 3, but with half the electrode spacing and, consequently, half the line length. Each line was acquired using two different electrode array configurations, dipole–dipole and gradient. Raw ERT data were inverted using open-source R2 version 4.0.2 ERT inversion software and the associated ResIPy version 3.3.2 software [44,45]. For consistency, the same inversion settings and starting resistivity were used for each inversion run. The mesh was a quadrilateral grid with increasing vertical cell size as the depth increased from the surface. The horizontal cell size was set such that there were 5 cells between each electrode (e.g., the horizontal cell size of 0.5 m for 2.5 m electrode spacing), regardless of the electrode spacing, again for consistency between the inversions. ResIPy inversions consider variation in surface topography through direct input of measured surface elevations. Depth of investigation, or the nominal location in the subsurface beyond which inversions lose accuracy, was calculated using the methods described in Oldenburg and Li [46], as incorporated into ResIPy. In addition to the two independent survey-type inversions for each ERT line, a combined inversion was also run on each line where the raw data from both surveys were inverted together, although R2 software limitations prevent the calculation of the depth of investigation in these combined inversions.

EM data were collected along walkable surface tracks between Last Chance and Goodenough lakes in October 2019 using a Geonics EM-31 (Geonics Limited, Missisauga, ON, Canada) instrument, which estimates the average electrical conductivity of the subsurface to depths of between 1 and 6 m, depending on the conductivity [47]. The vertical sampling provided by the EM31 is not sufficient to invert for vertical variations in the electrical properties; thus, the instrument only provides an estimate of the average conductivity over its depth of exploration. This limitation is balanced by its ease of operation, allowing rapid acquisition over large areas, which makes it a good complement to the more labor-intensive ERT methods.

### 2.4. Hydrogeologic Measurements

To provide context to the geophysical surveys and lake samples, climate analyses were performed based on measurements at the nearest climate station, 22 km to the southeast (10U 604590.33 m E 5666952.88 m N) over the period from 2016–2022 (Figure S1) [48]. Local hydrologic measurements were performed in piezometers installed in the hillslope adjacent to Last Chance Lake between fall 2021 and spring 2023. Continuous analyses of water table elevation, conductivity, and subsurface temperature were obtained in a piezometer (DP2) installed in the hillslope leading into the southwest corner of Last Chance Lake via a Solinst Levelogger^®^ 5 LTC at 5-min intervals; plotted values represent the 24-h running average of individual analyses. Manual water table measurements were obtained in both DP2 and a nearby piezometer installed closer to the lake, DP1, using a Heron Little Dipper water level meter.

### 2.5. Hydrogeologic Modeling

To build upon our geophysical snapshots and better understand the drivers and temporal stability of such concentrated alkaline waters, we simulated the hydrogeological dynamics of the Last Chance Lake subsurface using lake morphology derived from aerial imagery (Figure S6) and lake and groundwater compositions (Table S3) as inputs. The simulations calculate salinity-dependent fluid density and viscosity using a parameterized [49] and extensively tested [50] set of equations incorporated in the open-source reactive transport simulator PFLOTRAN [51]. Specifically, the Batzle and Wang [49] equation is used to calculate density and viscosity as a function of the equivalent NaCl salinity, which is, in turn, calculated from the actual measured concentrations of Na^+^, Cl^−^, Ca^++^, Mg^++^, SO_4_^−^, and HCO_3_^−^ input into the simulation (Table S3) in the initial instance, and updated as waters mix and concentrations evolve throughout the simulation. Boundary conditions and material properties used in the simulation considered observations of local hydrogeology and are described in detail in Section S3. Briefly, infiltration along the upper boundary occurred seasonally, with no infiltration during the 4 “winter” months and fluids infiltrating at a rate of 0.08 m/year for 8 months of the year. Infiltrating water chemistry was equivalent to a representative lake chemistry composition in the subsurface underlying Last Chance Lake and a representative groundwater composition (Table S3) elsewhere (Figure S6). Simulations were initialized using a hydrostatic boundary condition on upstream and downstream boundaries, with the surface of the downstream boundary set to atmospheric pressure (101,325 Pa) and the upstream (southwestern) boundary set to have 1 m higher hydraulic head, consistent with the regional hydraulic head gradient (Figure S5). The boundaries parallel to the flow and bottom boundary (at 20 m depth) were set to no-flow conditions, consistent with the limited extents of flow perpendicular to the head gradient and the inferred surficial hydrology. The 760 × 455 × 20 m domain was subdivided into 691,600 5 × 5 × 0.4 m elements, with the depth and tighter spacing in the z direction to capture the geophysically observed dynamics of vertical flow. All boundaries were set such that inflowing fluids maintain a constant chemical composition and outflowing fluids have zero diffusional gradients at the boundary.

## 3. Results

### 3.1. Geological and Hydrologic Setting of Alkaline Goodenough and Last Chance Lakes

Last Chance and Goodenough lakes share identical climate (Figure S1) and are fed by subsurface groundwater and its emanations as springs and seeps (Figures S2–S4; Renaut and Long [28]), minor direct precipitation (Figure S1), and snowmelt (Figure S5; Hirst [30]). Climate analyses since 2016 indicate annual rainfall in the region ranges from 243 mm (in 2022) to 412 mm (in 2016) and mean monthly temperatures range from −7.9 °C (December) and 16.8 °C (July) Figure S1 [39]. Typically, periods of elevated rainfall coincide with elevated monthly temperatures, but a substantial portion of the total precipitation occurs when temperatures are below freezing (Figure S1). As a result, our groundwater monitoring indicates a corresponding surge in the groundwater table during the spring snowmelt (Figure S5). Incoming groundwaters are transmitted into the topographical depressions that host both lakes through glacio-fluvial sands and gravels that overly demonstrate that pore waters near the spring are more dilute than those distal to the spring. Note that measurements of overlying lake waters were effectively homogeneous, indicating that the observed signal is exclusive to the subsurface.

**Figure 2:**
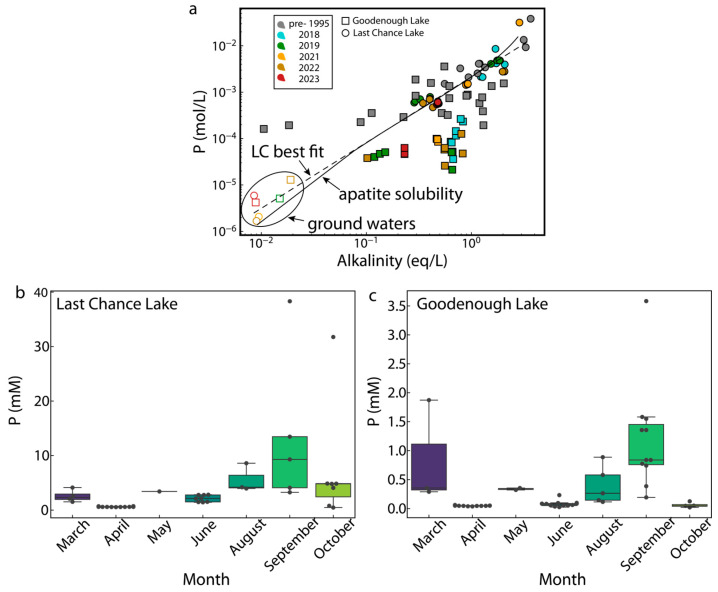
The groundwater–lake water divided into alkaline lake systems. (**a**) Phosphorus (P) and alkalinity measured in Last Chance and Goodenough Lake and groundwaters between 1991 and 2023. The dashed line is fit to the post-2018 Last Chance Lake data measured in this study, while the solid line represents calculated apatite solubility. The close agreement indicates apatite solubility likely controls total P concentration. Box and whisker plots of P concentrations as a function of the sampling month in (**b**) Goodenough Lake and (**c**) Last Chance Lake.

Tertiary basalts [28]. Our new measurements and those of Hirst [30] indicate that the chemical compositions of groundwaters and groundwater-derived springs and seeps feeding the lakes are very similar (Figure 2), yielding the Na-CO_3_-(SO_4_)-Cl chemistry common to both upon evaporation.

**Figure 3:**
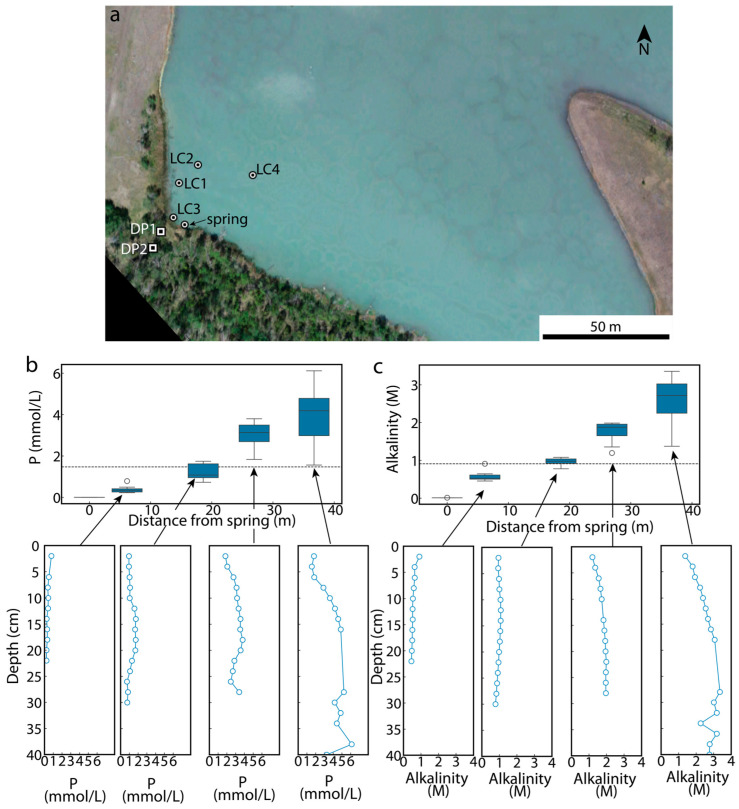
Demonstration of freshwater input on lake subsurface chemistry. (**a**) Drone imagery from June 2021 showing the location of a spring emanation (mapped in October 2019), the location of four sediment cores acquired from the lakebed, and the locations of installed piezometers. (**b**,**c**) Box and whisker plot of phosphorus concentrations (**b**) and alkalinities (**c**) measured in acquired cores as a function of distance from the spring.

Last Chance Lake waters are generally more concentrated than those in neighboring Goodenough (Figure 2; Data Set S1), and Last Chance tends to dry out completely each year while Goodenough does not (Figures S2–S5). Long-term wet–dry and freeze–thaw cycling at Last Chance has generated semicircular pools on the lake floor that decrease in diameter near the edges. Goodenough has no brine pools, although it does develop a salt crust around its outer edge late in the year (Figure S6). Aerial imaging (Figure S4b), field reconnaissance, and prior studies [30] demonstrate the presence of groundwater springs in the southwest corner of the lakes. Porewater measurements acquired from sediment cores obtained in the region near Last Chance Lake springs in June 2021 indicate that groundwater inputs dilute subsurface chemistry, with the effect diminishing approximately 20 m inward of the spring emanation (Figure 3).

While both lakes exhibit P concentrations amongst the highest ever measured in natural waters (Figure 2; [3,10]), and these concentrations increase (Figure 2b,c) as seasonal precipitation wanes (Figure S1) and water input to the lake diminishes (Figure S5), Last Chance’s fall season maximum alkalinity and P concentrations exceed Goodenough’s by over an order of magnitude (Figure 2). Insoluble apatite-group minerals (e.g., hydroxylapatite, Ca_5_(PO_4_)_3_OH) preclude high P concentrations in nearly all natural waters, but the development of alkaline conditions and the coupled sequestration of Ca in carbonate minerals dictates that apatite-group minerals are far more soluble in alkaline lakes like Goodenough and Last Chance than in typical, less carbonate-rich waters. Indeed, our geochemical calculations demonstrate that Last Chance Lake P concentrations can be predicted by simply assuming an alkaline lake water in equilibrium with the atmosphere and the minerals hydroxylapatite and calcite (Figure 2a). Goodenough Lake P concentrations tend to deviate from conservative behavior, likely due to the differing, and perhaps more productive, biota that inhabit it [10,35], but its peak concentrations may also be predicted thermodynamically (Figure 2a). This result suggests that any deviations in P concentrations in a prebiotic alkaline lake environment would be the result of lake water dilution or enhanced evaporation. In other words, the more concentrated nature and consequent higher alkalinity of Last Chance Lake are the main drivers of its higher peak P concentrations. *What then drives Last Chance Lake to become more concentrated than Goodenough Lake, and what are the implications of interactions between influxing, dilute groundwaters and dense, concentrated lake waters?* Geophysical observations can help to answer these questions, and, in turn, provide a better understanding of the coupled physical and chemical dynamics of evaporating alkaline lakes.

### 3.2. Geophysical Imaging of the Alkaline Lake Subsurface

**Figure 4:**
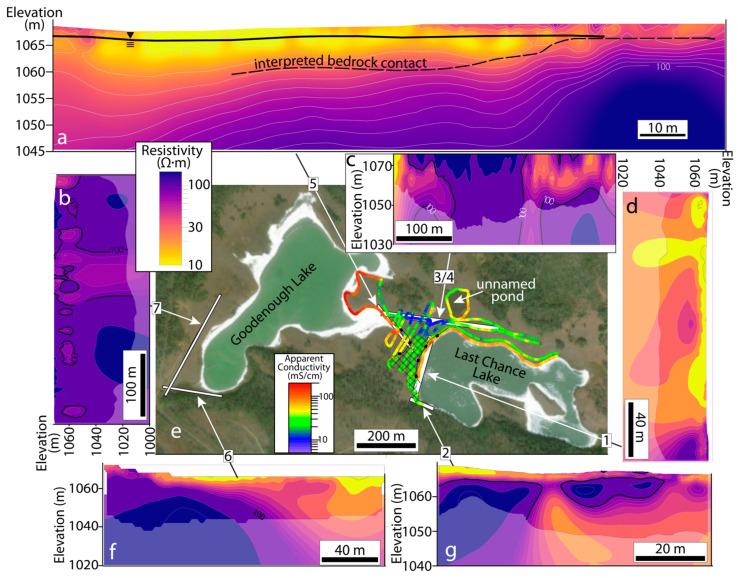
Geophysical investigations of the subsurface surrounding Last Chance and Goodenough Lakes are displayed in their spatial context. Electromagnetically (EM) measured apparent conductivity is overlain directly on the aerial imagery (**e**), while the locations of the seven numbered Electrical Resistivity Tomography (ERT) surveys (**a**–**d**,**f**,**g**) are shown in the surrounding area. The depth of Investigation in the ERT inversions is shown with a translucent overlay.

**Figure 5:**
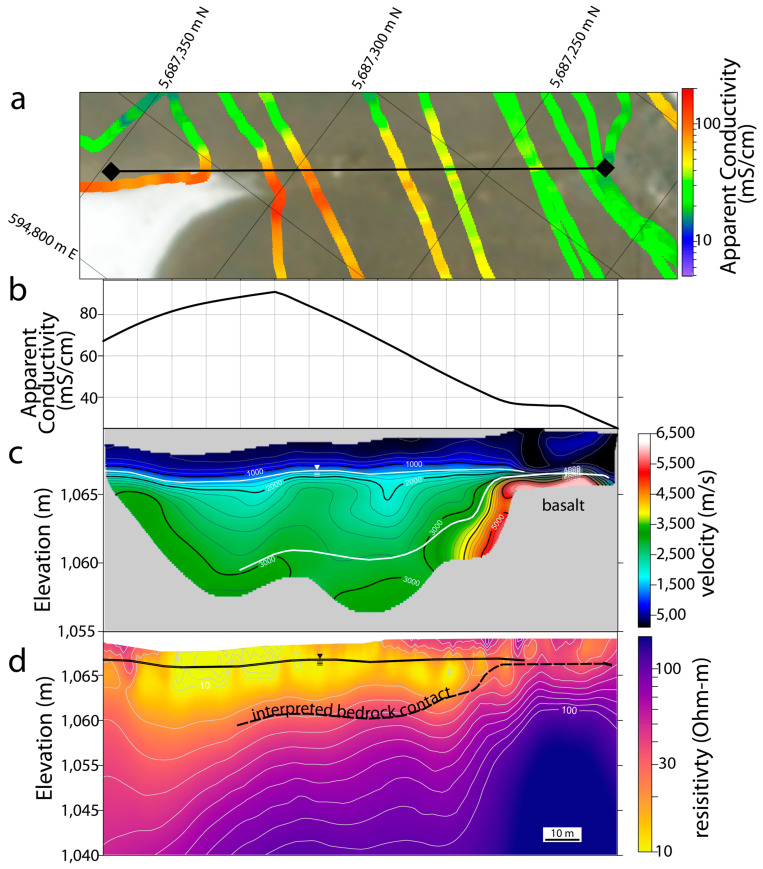
Geophysical survey results and interpretation from the between-lake survey (Line 5). (**a**) Site map showing the limits of the line extending from Last Chance Lake on the right (0 m) to Goodenough Lake on the left (150 m), with the subset of the electromagnetic (EM31) survey that intersects Line 5 overlain. (**b**) The apparent conductivities from the EM31 survey over Line 5. (**c**) The subsurface seismic velocities from the seismic refraction survey. (**d**) The modeled resistivities from the ERT survey. The top of the water table, interpreted at approximately 1500 m/s in the refraction survey, a value consistent with saturated soil, is indicated in (**c**,**d**). Additionally, the contact between the basaltic bedrock and overlying glacio-fluvial sands and gravels, interpreted based on the results from all three surveys, is indicated in (**c**,**d**).

The results of our geophysical investigations elucidate the physical, chemical, and hydrogeologic properties of the subsurface underlying Last Chance and Goodenough Lakes (Figure 4). The seismic refraction survey traversing the land between the lakes shows clear contrasts in the velocity distribution (Figure 5). In these data, a low-velocity (<1500 m/s) zone appears in the uppermost ~3 m of the subsurface whose lower boundary mimics the overlying surface topography. Further into the subsurface, a region characterized by seismic velocities of ~2000–3500 m/s begins at the lower boundary of this low-velocity region and continues to the lower boundary of the investigated region, ~10 m into the subsurface, throughout most of the subsurface. However, on the Last Chance Lake side of this seismic line, a high-velocity zone (>4000 m/s) replaces this 2000–3500 m/s zone that otherwise characterizes these depths of the subsurface.

Electrical Resistivity Tomography (ERT) and electromagnetic (EM) surveys complement physical property characterization provided by the seismic refraction survey by interrogating the electrical properties of the subsurface. Resistivities of the subsurface underlying the southern shores of both lakes (Figure 4f,g) indicate highly conductive fluids overlying a lens of more resistive (i.e., fresher) groundwaters. Consistent with pore water measurements (Figure 3), the influence of influxing groundwater on the subsurface resistivity diminishes by ~30 m lakeward as the subsurface conductivity is increasingly dominated by conductive lake-derived brines (Figure 4d), which control the average apparent conductivity of the shallowest subsurface along both lakes (Figure 4e). A continuous parcel of high-conductivity subsurface brine leading from the higher elevation of Goodenough Lake towards the lower elevation of Last Chance Lake also reveals subsurface hydrological communication and solute transport between the two lakes (Figure 4a,e). In numerous locations (Figure 4c,d,f,g), continuous, high-conductivity brine features appear to drip from the lake downwards into the fresher subsurface.

### 3.3. Dynamics of Surface–Subsurface Interactions

**Figure 6:**
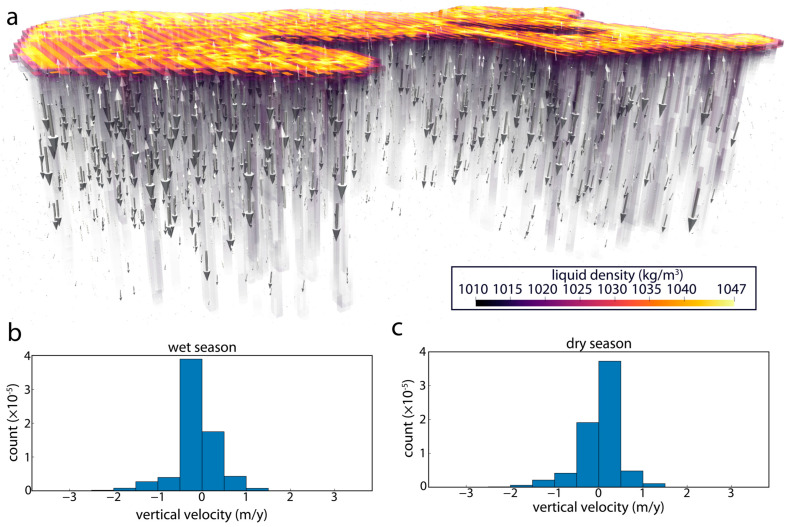
Hydrogeologic modeling surface–subsurface interactions at Last Chance Lake. (**a**) Hydrogeologic simulations of groundwater-surface water interactions at Last Chance Lake, showing fluid density and velocity vectors in the subsurface after the simulation reached pseudo-steady state. Histograms of the vertical (z) component of fluid velocity during the wet season (**b**) and dry season (**c**) illustrate the rate and extent of downward and upward fluid movement in the alkaline lake subsurface. Note the domain is vertically exaggerated by a factor of 10.

**Figure 7:**
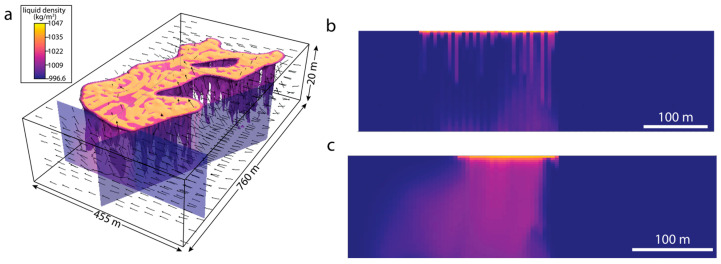
Hydrogeologic simulations of groundwater-surface water interactions at Last Chance Lake. (**a**) Visualization of the full simulated domain after reaching pseudo-steady-state flow configuration, showing velocity vectors proportional to their magnitude (range 0.01–5.6 m/y) and fluid density as isosurfaces and cross-domain slices. (**b**) Subsurface fluid density along the southwestern shore, showing the region interrogated with ERT line 2 (Figure 4g). (**c**) Subsurface fluid density along the western shore, showing the region interrogated with ERT line 1 (Figure 4d). The domain has also been vertically exaggerated (see annotations in a). Although fluid density is not a primary contributor to electrical resistivity, the density model used here integrates charged ion concentrations to calculate density in a manner that mimics the effect of ion concentration on fluid resistivity.

Despite the simplicity of the parameterization, the hydrogeological simulation results (Figure 6 and Figure 7) exhibit striking agreement with the geophysical surveys. The agreement is particularly evident when comparing observed (Figure 4 and Figure 5) and simulated (Figure 6 and Figure 7) subsurface locations of brines and groundwaters. In particular, the ERT and EM31 observations and simulated results both indicate an elevated concentration of high-density brines near the ground surface where they are generated through evaporative processes. Moreover, the narrow (<5 m) fingers of infiltrating brine along the lake’s southern shore observed in the ERT surveys (Figure 4g) are consistent with the distribution of dense brine in the subsurface hydrogeologic simulations (Figure 7), in which their infiltration is driven by density instabilities and the resultant convective motion. Finally, the increasing dominance of brines over fresh groundwaters in the subsurface lakeward of the infiltrating groundwater measured in pore water profiles (Figure 3b,c) and observed in the ERT survey (Figure 4a,d and Figure 5) is matched by the simulated distribution of brine in the Last Chance Lake subsurface (Figure 7c).

These hydrogeological calculations also reveal the unexpected dynamical behavior of this concentrated alkaline lake system that explains patterns of brine infiltration observed in the ERT data. In the simulation, density contrasts between groundwater and brine create Rayleigh–Taylor instabilities, which, in turn, drive vigorous vertical circulation of subsurface fluids. This density-driven flow leads to a dynamic exchange of fluids between the surface and subsurface (Figure 6) and mixing between groundwaters and infiltrating lake water brines (Figure 7). The strength of the convection at the groundwater–lake water interface underlying the lake shores drives tighter convection cells and enhanced mixing in the subsurface underlying the lake’s edge, co-located with the smaller, more closely packed pools along the lakeshore (Figure S4b).

## 4. Discussion

### 4.1. Subsurface Structure and Hydrogeology

Interpretation of the subsurface velocity distribution derived from the seismic refraction data reveals a transition from unobservable basaltic bedrock in the ~10 m of subsurface sediments underlying Goodenough Lake to clearly defined basalts ~5 m under Last Chance Lake. Geomorphological processes—i.e., those that eroded the bedrock surface and deposited the basalt-mantling sediments—have thus resulted in a reduced lake depth and a reduced cross-sectional area through which fresh groundwaters can feed into Last Chance and compared to neighboring Goodenough Lake. A direct consequence of this subsurface structure is that, despite their identical climate, the differing geomorphology of the two lakes leads Last Chance to evaporate to dryness each year, while Goodenough persists as a perennial lake. As noted above, maximum concentrations in both Last Chance and Goodenough Lakes at a given alkalinity (or degree of evaporation) can be predicted from simple calculations of hydroxylapatite solubility (Figure 1). Moreover, since the input fluid chemistry to both lakes is the same (Figure 1), our results thus demonstrate that the reason Last Chance Lake has the highest phosphorous concentrations observed in any natural waters—concentrations that are an order of magnitude higher than neighboring Goodenough—is simply its geomorphologically driven tendency to dry up each year.

Together, geochemical measurements of groundwaters, lake waters, and pore waters (Figure 2 and Figure 3), geophysical surveys (Figure 4 and Figure 5), and hydrogeologic measurements (Figures S1 and S5) confirm that groundwater influx serves as the principal source of solutes and water to the lakes, consistent with a recent analysis of the mass balance of phosphorus in these lakes [39]. Our field investigations show that the essential but limited groundwater flux into the lakes is chemically overwhelmed by the more volumetrically abundant surface and subsurface brine, such that the seasonal influx of waters during the spring period (Figure S2) cannot dramatically freshen the lake waters. These observations are consistent with chemical analyses (Figure 2b,c) and the timescales of thousands of years required to reach the seasonally elevated phosphorus concentrations Last Chance Lake contains today [39]. In turn, these observations suggest that the unique subset of shallow, alkaline lakes that form in small, closed-basin, low-relief catchments—of which Last Chance Lake is the only currently identified example [3]—most readily achieve extreme P enrichments due to their unique balance of limited groundwater input and significant evaporation. Higher ratios of evaporation to groundwater influx would transform these lakes into perennial salt flats, whereas lower ratios would drive their dilution (e.g., in the example of Goodenough Lake). This requirement of tightly balanced ratios of inflow to evaporation implies that changes to regional climate can substantially impact a particular alkaline lake’s astrobiological potential.

Because density contrasts drive vigorous fluid exchange and mixing, our transport modeling results imply that both the hydrogeologic setting that has promoted the development of concentrated brine, and the presence of the brine itself, underpins dynamic surface–subsurface fluid convection and solute transport. For example, if fresher groundwater influx were muted or stopped, or the subsurface did not attain the elevated permeability required for density-driven convection (>1 × 10^−14^ m^2^; [52]), exchange would be limited and likely restricted to unidirectional, dense brine infiltration into the subsurface. In this case, resurfacing is nevertheless possible if the brine moves downgradient towards other lakes (cf. Figure 4). Because alkaline conditions are generally only attained through extensive evaporative concentration of dilute groundwater inflow, these phenomena are likely inherent to the generation and stabilization of alkaline lakes more broadly, in particular those that are concentrated enough to satisfy chemical criteria imposed by prebiotic synthesis experiments.

### 4.2. Prebiotic Compatibility and Habitability in Alkaline Lake Environments

**Figure 8:**
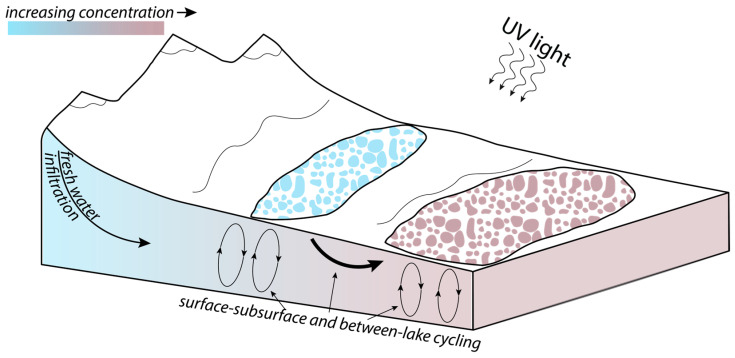
A revised conceptual model for alkaline lake systems as prebiotic environments. Contrary to our previously limited understanding of alkaline lakes derived from spatially decoupled measurements of brine chemistry evolution, our physical and chemical observations allow us to put forth this holistic model, in which waters cycle between the surface and subsurface, and between fresh and concentrated waters. In this way, the complex physical and chemical processing inherent to prebiotic alkaline lake environments thus may have not only facilitated prebiotic reaction networks, but also provided habitable environments for the earliest evolution of life.

Although alkaline lakes offer several distinct advantages for prebiotic synthesis [2,3,10,26,39,53], the conflicting effects of factors such as UV light, salinity, metal ion concentration, and pH have remained a challenge to reconcile within one environment. Our results show that the alkaline lake environments that may have facilitated the synthesis of multiple molecular building blocks were inherently subject to complex physical and chemical processing. Most importantly, surface–subsurface fluid convection within alkaline lake environments would have established strong vertical and lateral chemical heterogeneity, allowing chemical components synthesized in one portion of the aqueous system (e.g., through UV-promoted HCN homologation chemistry or carboxysulfitic chemistry at high phosphate concentration) repeated access to large gradients in pH and metal ion concentration, exposure to photic and aphotic regions of the hydrogeologic system, contact with porous substrates hosting subsurface fluids, and the potential for nascent organisms to expand into and colonize neighboring water bodies via subsurface transport (Figure 8). Although this greatly expands the complexity of prebiotic alkaline lake environments, coupling geological and geophysical observations with hydrogeochemical simulations provides initial steps toward a quantitative physico-chemical framework to investigate the subsequent processing of newly synthesized chemical components. More broadly, the results show that the chemical evolution of both modern and ancient alkaline lakes on Earth, and on ancient Mars, cannot be understood without explicit consideration of the physical processes that shape them.

## 5. Conclusions

While alkaline lakes are generally thought to have facilitated prebiotic synthesis reactions on the early Earth, the conditions required for some building block synthesis reactions are distinct from others, and these conditions are generally incompatible with those permissible for nascent cellular function. However, our knowledge of whether these environments can overcome these potential limitations has been limited, because an integrated investigation of the physical and chemical features that characterize these environments has so far been lacking from the astrobiological literature. Here, we investigate the dynamics of active, prebiotically relevant Last Chance and Goodenough Lakes in British Columbia, Canada using near-surface geophysics, aqueous geochemistry, and hydrogeologic modeling. Our results show that, although both Last Chance and Goodenough Lakes are fed by groundwater with the same composition, the root cause of the order-of-magnitude higher phosphorus concentrations in Last Chance Lake relative to neighboring Goodenough Lake is its geomorphologically-driven vulnerability to evaporation. The resultant density contrast between evaporated brines and inflowing groundwaters yields Rayleigh-Taylor instabilities and vigorous surface–subsurface cycling and mixing of lake and groundwaters. In a prebiotic context, these processes would have allowed waters, solutes, and nascent organisms repeated access to large gradients in pH and metal ion concentration, exposure to photic and aphotic regions of the hydrogeologic system, contact with porous substrates hosting subsurface fluids, and the ability expand and colonize environments. Thus, the complex physical and chemical processing inherent to prebiotic alkaline lake environments may have not only facilitated prebiotic reaction networks, but also provided habitable environments for the earliest evolution of life.

## Data Availability

All data except for raw geophysical data are available in the main text or the Supplementary Materials. Raw geophysical data are available from the authors upon request. ERT data were processed using open-source R2 ERT inversion software and the associated ResIPy software (https://gitlab.com/hkex/resipy, accessed on 10 November 2024). The Rayfract^®^ software package used in processing seismic refraction data is a proprietary software package that can be licensed from Intelligent Resources, Inc. EM31 data were processed using associated software provided by Geonics Limited.

## Supplementary Materials

The following supporting information can be downloaded at: https://www.mdpi.com/article/10.3390/life14121624/s1.
