# Supplementary material for: Groundwater-Driven Evolution of Prebiotic Alkaline Lake Environments
Source: Life (Basel). 2024 Dec 7;14(12):1624. doi: 10.3390/life14121624 (PMC11678467; doi:10.3390/life14121624)
Supplement: Supplementary file 1 [file life-14-01624-s001.zip › Tutoloetal_SI.pdf]

Supplementary Materials for

Groundwater-Driven Evolution of Prebiotic Alkaline Lake Environments

Benjamin M. Tutolo [1,*], Robert Perrin [1], Rachel Lauer [1], Shane Bossaer [1], Nicholas J. Tosca [2], Alec Hutchings [1], Serhat Sevgen [1], Michael Nightingale [1], Daniel Ilg [1], Eric B. Mott [1] and Thomas Wilson [1]

[1] Department of Earth, Energy, and Environment, University of Calgary, Calgary, AB T2N 1N4, Canada

[2] Department of Earth Sciences, University of Cambridge, Cambridge CB2 3EQ, UK

***Correspondence: benjamin.tutolo@ucalgary.ca**

This PDF file includes:

Supporting text
Figures S1 to S6
Tables S1 to S3
Legends for Datasets S1 to S2
SI References

Other supporting materials for this manuscript include the following:

Data S1 to S2

S1. Lake and groundwater Chemical Analyses

Field parameters (location, pH, temperature, and electrical conductivity) were recorded as soon as possible after sampling. The obtained lake, spring, or ground water samples were filtered through 0.45 μm polyvinylidene fluoride (PVDF) filters, and either acidified using strong acid (10% HCl) for cation analyses via Inductively Coupled Plasma Optical Emission Spectroscopy, diluted using deionized water (18 MΩ) for anion analyses via Ion Chromatography, or retained in filtered, raw form for alkalinity analyses. Preserved samples were all stored in acid-washed, tightly sealed bottles or tubes. Dilutions were calculated using recorded acid or DI water mass and the final sample mass. Alkalinity was measured via titration with 0.02 N H_2SO_4 on a Thermo Scientific Orion Star™ T910 autotitrator, typically on diluted raw samples within several days of sampling, but, in some instances, was subsequently (re-)analyzed on the deionized water-diluted "anions" sample. Phosphorus concentrations were occasionally measured with IC techniques, but analyses were most often performed spectrophotometrically using an automated Thermo Scientific™ Gallery™ method (analytical method is labeled in the data table). This method reacts orthophosphate ions in the sample with ammonium molybdate and an antimony potassium tartrate catalyst under acidic conditions to form a 12-molybdophosphoric acid complex, which is then reduced with ascorbic acid and measured spectrophotometrically. The sample's orthophosphate concentration is then calculated from the measured absorbance using a calibration derived from identical measurements performed on standards of known concentration. Repeat, spatially distributed samples were acquired on 24 April 2023 to confirm the limited spatial variability of P concentration; these were all assumed to have the same alkalinity, which was measured on just one of the samples. We supplemented our analyses with those reported by Hirst [1] and compiled by Toner and Catling [2]. As a check on the chemistry determinations, the collected analyses were filtered to exclude samples whose charge balance error (CBE), calculated according to:

$$CBE = \frac{|\sum cation * valence| - |\sum anion * valence|}{|\sum cation * valence| + |\sum anion * valence|} * 100\%$$

was greater than 10%, except in the few instances where there was a clear reason for this - e.g., Na^+ or alkalinity was not analyzed. This screening procedure resulted in a total of 49 and 54 lake and groundwater samples from Last Chance Lake and Goodenough Lake, respectively.

S2. Pore water acquisition and analysis

We extracted four sediment cores from Last Chance Lake in June 2021 with varying distance from the site of observed groundwater springs. Though we

initially sought a linear transect, a thick layer of predominantly sodium carbonate crystals made coring impossible in certain locations. Sediment cores were extracted in PVC core liners using a homemade piston corer. The sediment-water interface was preserved by adding sodium polyacrylate to the overlying lake water captured in the core tube immediately after collection. Porewaters were extracted from sediment cores at 2 cm intervals below the sediment-water interface using Rhizon samplers (Rhizophere Research Products) inserted into holes drilled directly into the core liner within 24 hours of sediment core collection. A large buildup of salt crystals from 18–28 cm depth in core LC4 prevented Rhizons from being inserted into the sediment. Alkalinity was determined on porewater samples using a Thermo Scientific OrionStar T910 autotitrator with a Ross Ultra pH electrode (8157BNUMD) calibrated with NIST-traceable pH 4, 7, and 10 buffers. For each titration, 1 mL of sample was diluted in ~99 mL of deionised water and titrated with 0.2 M H_2SO_4. Repeat measurements of a 0.45 M bicarbonate standard resulted in a 2 σ standard deviation of 0.005 M (n = 26). Phosphate concentrations were determined using IC methods described above. We plot analyses as a function of distance from a spring emanation point identified during complete lake dryout in June 2021 (Figure 2). Plotting the analyses as a function of distance from the lake shore would yield identical conclusions.

S3. Reactive transport simulations The Batzle and Wang [3] equation used in the reactive transport simulations calculates brine density from the density of pure water at the temperature and pressure conditions of interest using an empirical equation fit to measurements of NaCl brine densities. Adams and Bacchu [4] evaluated the efficacy of a series of equations, including the Batzle and Wang [3] parameterization, for calculating the density and viscosity of 4,584 sedimentary basin brines containing complex and varying mixtures of Ca^{++}, Mg^{++}, SO_4^{2-}, HCO_3^-, CO_3^{-2}, Na^+, K^+, and Cl^-, up to greater than 0.25 mass fraction equivalent NaCl. This analysis demonstrated that the Batzle and Wang [3] equation yields accurate calculations of complex brine densities, and, importantly, that the Batzle and Wang [3] equation outperforms calculations based on other potential brine compositions ($CaCl_2$, $NaSO_4$, and a 50% $CaCl_2$/NaCl mixture).

Boundary conditions and material properties used in the simulation considered observations of local hydrogeology. Regional groundwater flow is driven from the most significant topographical feature in the region, the Marble Range, ~20 km to the southwest [1]. Consistent with this regional groundwater flow, the southern shores of both lakes are vegetated by dense, groundwater-fed conifer forest right to their edge, while their northern shores are populated solely by grasses at some distance from the lake edge (Figs. 3e, S2-S4). The estimated subsurface intrinsic permeability of 1×10^{-12} m^2 applied throughout the domain was

calculated from the hydraulic conductivity determined by applying the Hvorslev method to measurements of the hydrologic head rebound in a drive point piezometer (DP2) located 20 m shoreward of the southwest shore of Last Chance Lake (Figure 2) following complete bailout for sampling on November 14, 2021, April 3, 2022, and June 21, 2022. While authigenic minerals formed in Last Chance and Goodenough Lake tend to be fine-grained, the shallow subsurface is dominated by glacial material with much larger typical grain sizes [1], which gives confidence to this relatively high estimated permeability. Simulations were run in parallel on up to 192 cores on the University of Cambridge CSD3 cluster, output as HDF5 files, and visualized in Paraview (Figure 5; www.paraview.org/) and VisIt (Figure 6; https://visit-dav.github.io/), which also permitted calculation of streamlines from outputted flow velocities.

References

1. Hirst, J.F. Sedimentology, Diagenesis and Hydrochemistry of the Saline, Alkaline Lakes on the Cariboo Plateau, Interior British Columbia, Canada, University of Saskatchewan, 1995.
2. Toner, J.D.; Catling, D.C. A Carbonate-Rich Lake Solution to the Phosphate Problem of the Origin of Life. *Proc. Natl. Acad. Sci. U. S. A.* **2020**, *117*, 883–888, doi:10.1073/pnas.1916109117.
3. Batzle, M.; Wang, Z. Seismic Properties of Pore Fluids. *Geophysics* **1992**, *57*, 1396–1408, doi:10.1190/1.1443207.
4. Adams, J.J.; Bachu, S. Equations of State for Basin Geofluids: Algorithm Review and Intercomparison for Brines. *Geofluids* **2002**, *2*, 257–271, doi:10.1046/j.1468-8123.2002.00041.x.

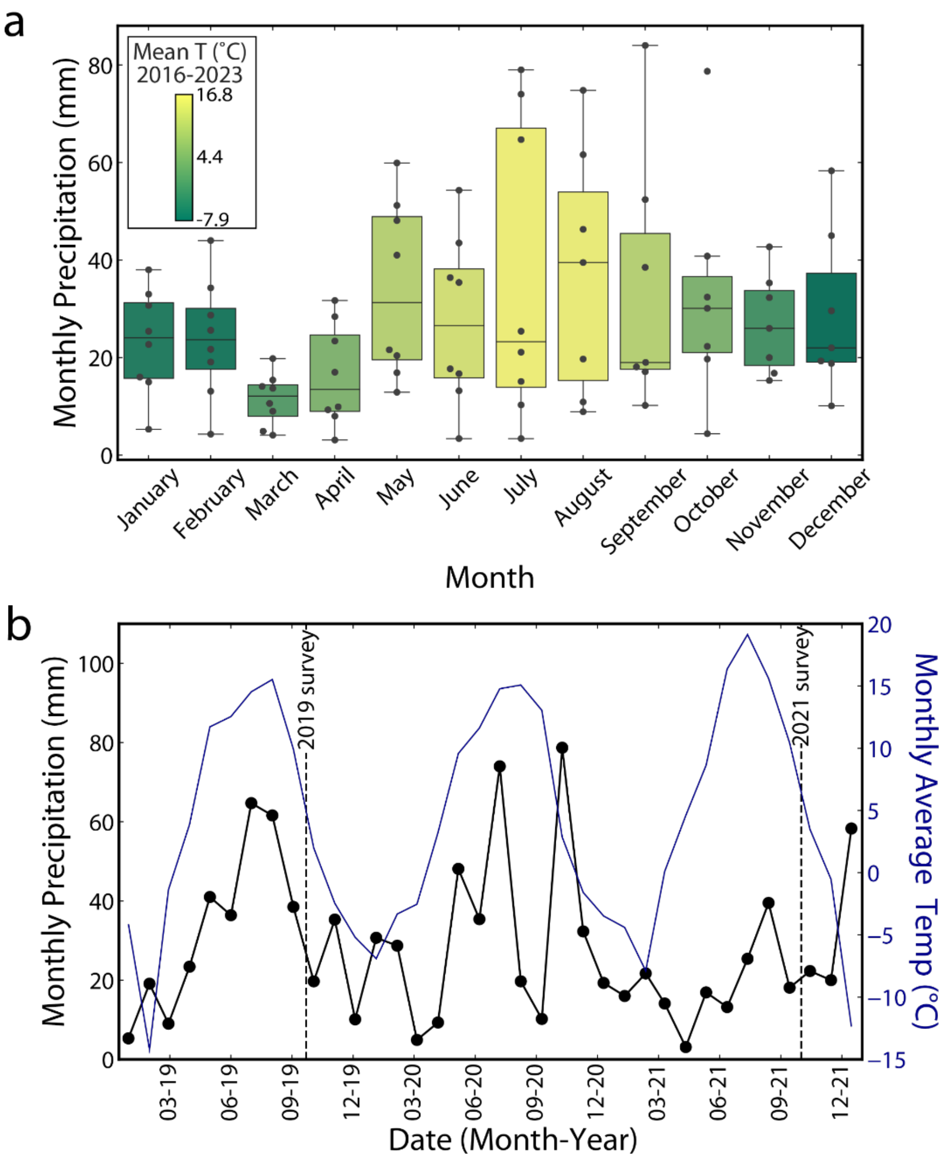

Figure S1. (a) Monthly precipitation recorded at the Clinton RCS weather station, with boxes colored to represent mean monthly temperature. **(b)** Monthly precipitation and average temperature from January 2020 to December 2021. Geophysical surveys were performed in October 2019 and October 2021.

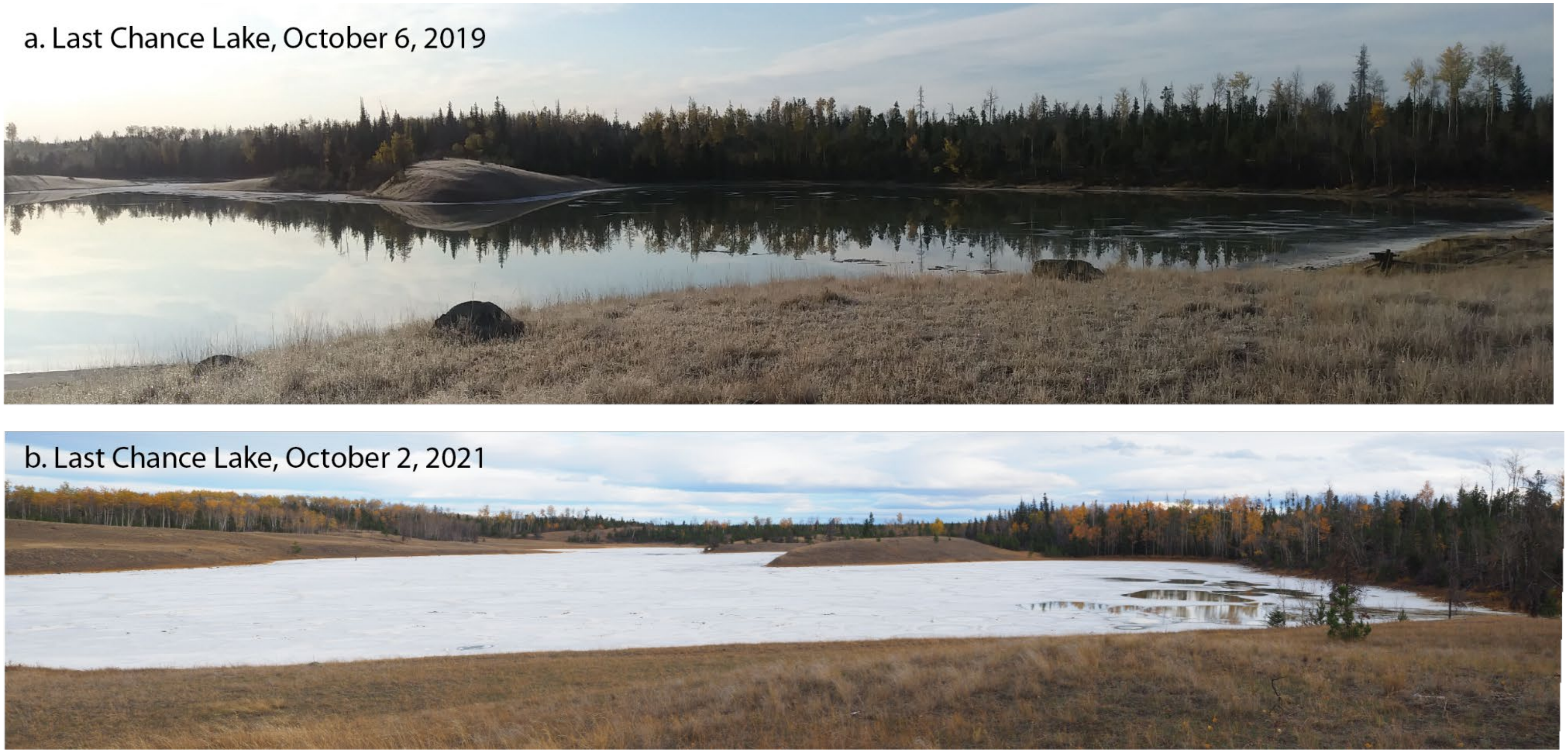

Figure S2 Photographs of the southern portion of Last Chance Lake, taken in **(a)** October 2019 and **(b)** October 2021. Water on the surface in the lower right of **(b)** occurs in a region of known groundwater emanations.

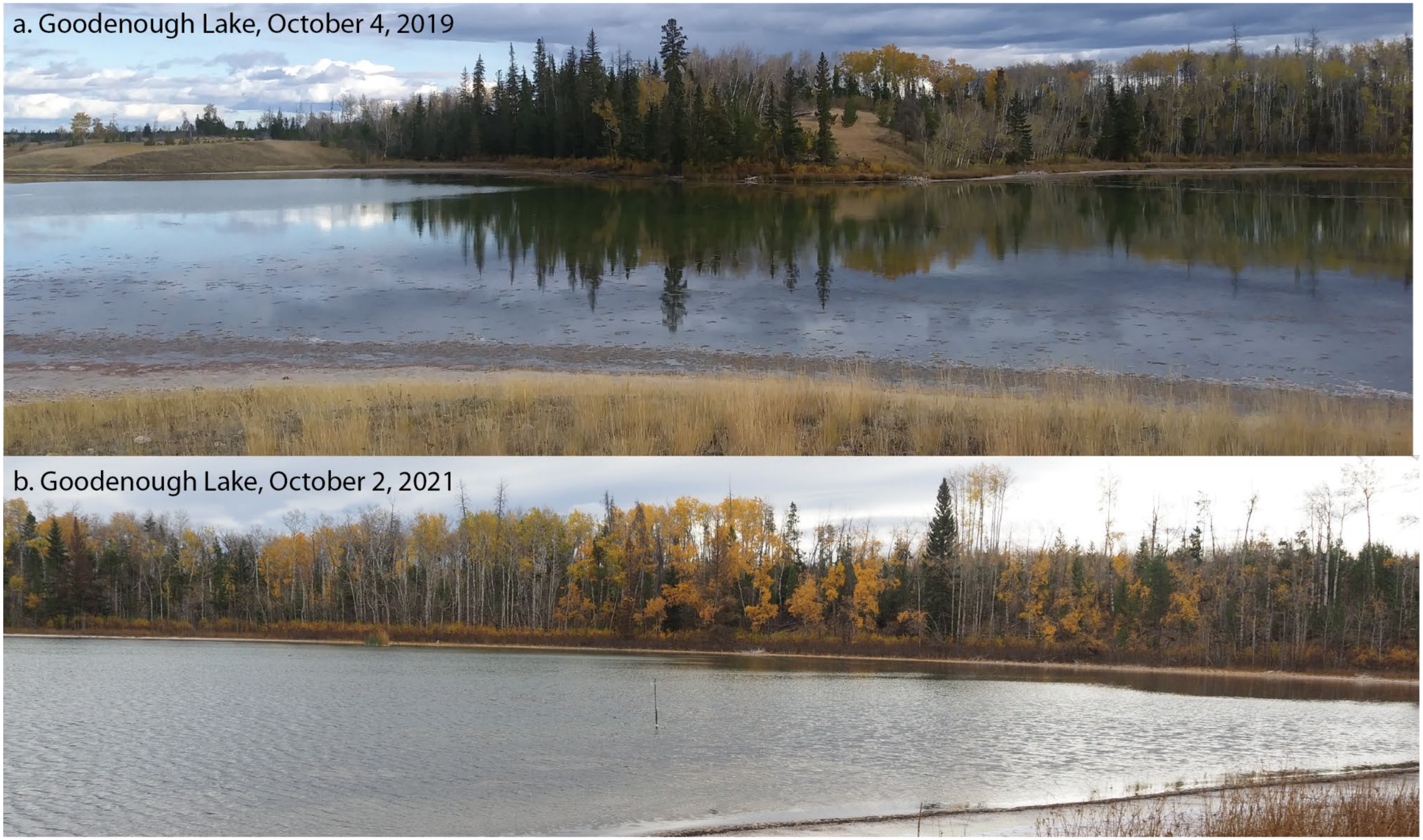

Figure S3. Photographs of the southern portion of Goodenough Lake, taken in **(a)** October 2019 and **(b)** October 2021.

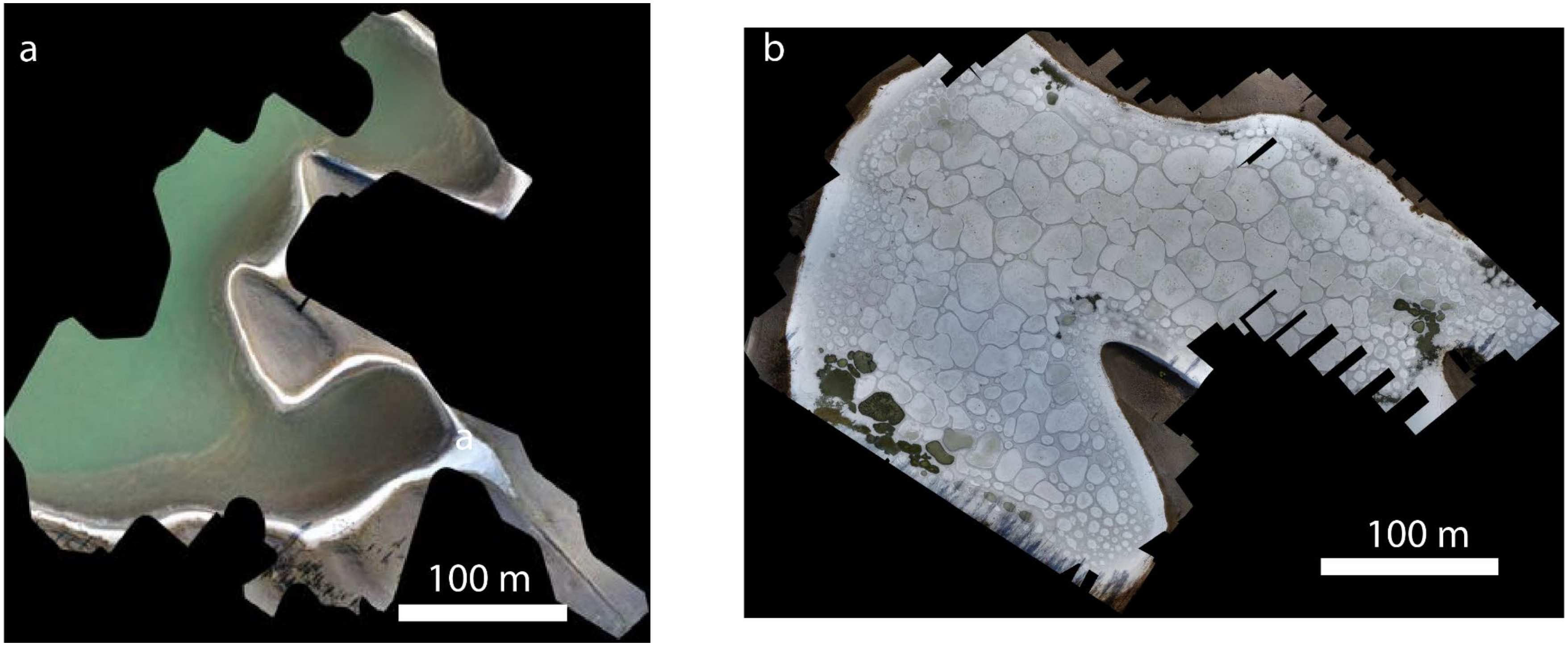

Figure S4 Aerial images of **(a)** Goodenough Lake and **(b)** Last Chance Lake acquired on the same day in October 2021 and demonstrating the contrasting end-of-season dry out behavior between the lakes. Water on the surface in the lower left of **(b)** occurs in a region of known groundwater emanations (see Figure S2b).

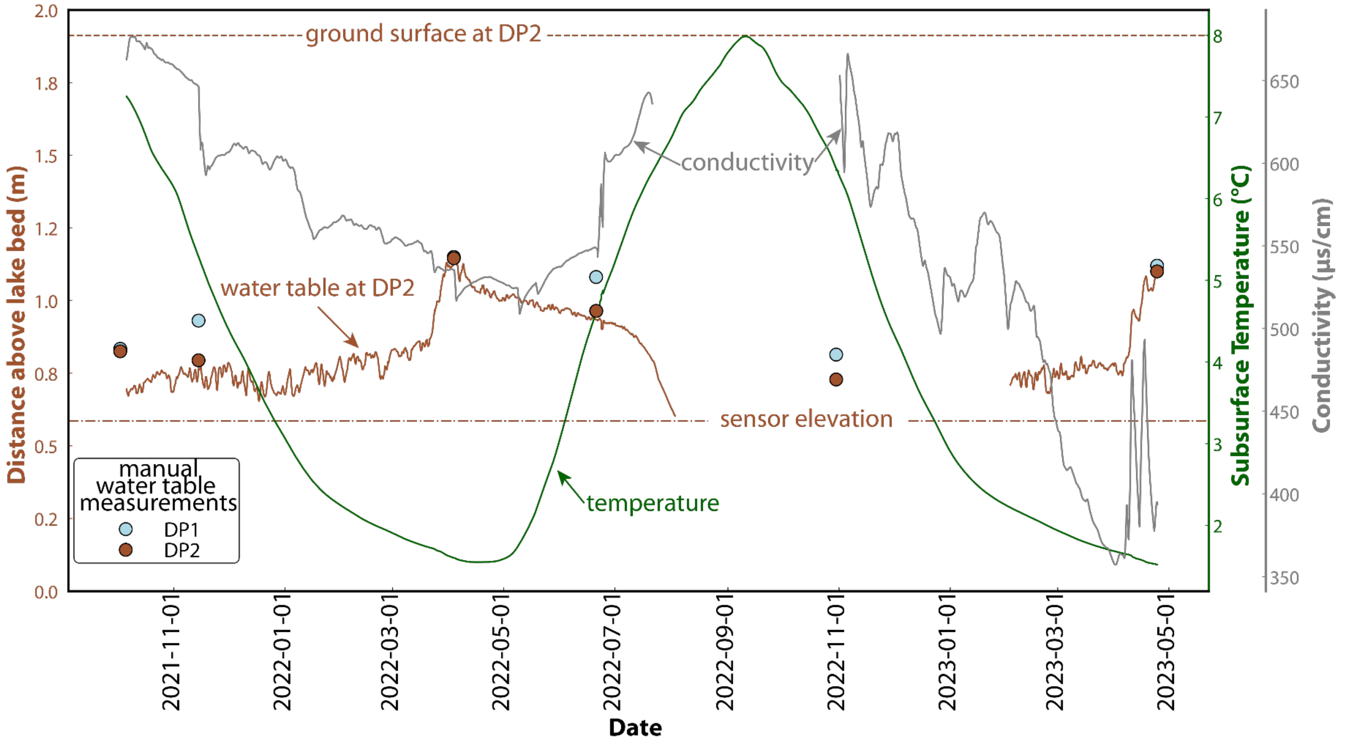

Figure S5. Hydrologic measurements performed in the hillslope adjacent to Last Chance lake (see Figure 2 for locations) between fall 2021 and spring 2023. Elevations are referenced to the elevation of the lakebed measured in October 2019. Continuous analyses of water table elevation, conductivity, and subsurface temperature were obtained via a Solinst Levelogger® 5 LTC at 5 minute intervals; plotted values represent the 24-hour running average of individual analyses.anual water table measurements were obtained using a Heron Little Dipper water level meter. Falling levels in the piezometer in summer 2022 led to unreliable measurements and these data were therefore not plotted.

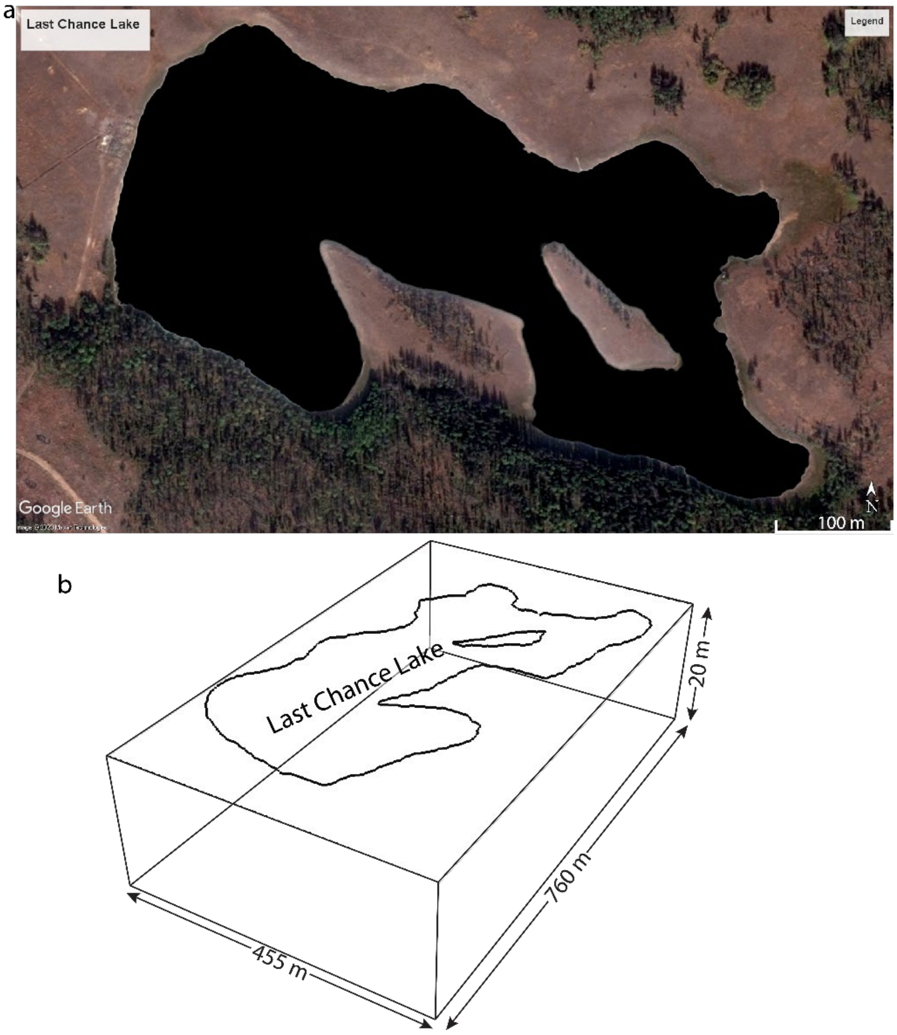

Figure S6 (a) Mask used to set upper boundary conditions in PFLOTRAN simulations of groundwater-lake water interactions at Last Chance Lake. (**b)** Schematic representation of the simulated domain (note 10× vertical exaggeration). Boundary conditions and discretization are described in Methods. Water infiltrating the masked lake surface had the composition of the lake water while water infiltrating the surrounding ground surface and the upstream (south) boundary face had the composition of the groundwater.

Table S1 Locations, mean, and standard deviation of lake water, pore fluid, and ground water alkalinity and phosphorus analyses plotted in Figure S2. All data are given in DataSet S2.

					Alkalinity		Phosphorus	
**Name**	**UTM 10U N**	**UTM 10U E**	**Distance from spring emanation (m)**	**Maximum sampling depth (cm)**	**Mean (M)**	**σ (M)**	**Mean (mM)**	**σ (mM)**
Last Chance Lake[a]	-	-	-	-	0.91	0.02		
LC1	5687074	594946	18.6	30	0.97	0.09	1.21	0.37
LC2	5687082	594953	26.93	28	1.76	0.26	3.00	0.60
LC3	5687060	594943	6.16	20	0.57	0.13	0.38	0.17
LC4	5687078	594977	36.71	40	2.58	0.56	3.81	1.38
Spring[b]	5687056	594948	0	-	0.0100	0.0015	0.0032	0.0023

[a]The reported values for Last Chance Lake are representative of those obtained on June 7, 2021, at the time of core acquisition.

[b]Spring location is given as the measured emanation point determined in October 2019, while the reported spring water alkalinity values correspond to all Last Chance groundwater samples obtained between Jun 2021 and Apr 2023

Table S2 ERT survey electrode spacings and line lengths

Line	Number of electrodes	Spacing (m)	Line Length (m)
1	81	2.5	200
2	81	1	80
3	81	5	400
4	81	2.5	200
5	81	2.5	200
6	81	2.5	200
7	81	5	400

Table S3 Parameters used in the reactive transport simulations

**Fluid Chemistry**		
	lake water[1]	ground water[2]
pH	10	7.48
HCO_3^- (mol/L)	1.98	0.0094
Na^+ (mol/L)	2.31	0.00485
Cl^- (mol/L)	0.109	0.00026
SO_4^{--} (mol/L)	0.149	0.00058
Ca^{++} (mmol/L)	0.15	0.84
K^+ (mmol/L)	16.4	0.15
Mg^{++} (mmol/L)	1.26	2.46
HPO_4^{--} (mmol/L)	2.77	0.0021
density (kg/m^3)	1115	996.1
**Hydrogeologic Properties**		
Permeability (m^2)	1×10^{-12}	
Porosity (-)	0.32	
Diffusivity (m^2/s)	1×10^{-9}	
Tortuosity	0.5	

[1]Sampled on 23 June 2021; [2]Sampled on 14 Nov 2021

Dataset S1 (separate file). Table of analyses plotted in Figure 1.

Dataset S2 (separate file). Table of analyses plotted in Figure 2.
